# Correction: McEvoy et al. Canine Socialisation: A Narrative Systematic Review. *Animals* 2022, *12*, 2895

**DOI:** 10.3390/ani13010081

**Published:** 2022-12-26

**Authors:** Victoria McEvoy, Uri Baqueiro Espinosa, Andrew Crump, Gareth Arnott

**Affiliations:** 1 Institute for Global Food Security, School of Biological Sciences, Queen’s University Belfast, 19 Chlorine Gardens, Belfast BT9 5DL, UK; 2 Centre for Philosophy of Natural and Social Science, London School of Economics and Political Science, Houghton St., Holborn, London WC2A 2AE, UK

The authors wish to make the following corrections to this paper [1]:

In the original article [1], the study by Wormald et al., 2016, was incorrectly interpreted. Corrections of the studies’ main findings are stated below. The authors apologize for any inconvenience caused and state that the scientific conclusions are unaffected. The original article has been updated.

## Text Correction

On page 10, the Main Findings section of the table for the reference Wormald et al., 2016:

“Early exposure of puppies in public areas not correlated with reduced inter-dog aggression in adult dogs”.

Should be revised to:

“Early exposure of puppies in public areas was negatively correlated with reduced inter-dog aggression in adult dogs”.

On pages 23–24, in the fourth paragraph of 4.3.2. Non-C-BARQ:

“Early exposure to public areas was not correlated with reduced inter-dog aggression as adults [98]”.

Should be revised to:

“They found that there was actually no protective advantage of earlier or more frequent public exposure on the development of aggression as adults. This was demonstrated by survey results, which showed that every week an owner waited to expose their puppy to public areas, the more reduced the odds were that the puppy would show aggressive behaviour towards unfamiliar dogs as an adult. This study highlights the importance of more research being carried out on how dogs are socialised to unfamiliar dogs in early life, as this study demonstrates that mere simple introductions, i.e., exposures to unfamiliar dogs in a public park, may actually have more deleterious effects on future behaviour and their ability to cope with stress, when compared to more controlled, structured introductions during events like puppy classes [64,98]”.

## Reference Correction

“98. Wormald, D.; Lawrence, A.J.; Carter, G.; Fisher, A.D. Analysis of correlations between early social exposure and reported aggression in the dog. *J. Vet. Behav*. **2016**, *15*, 31–36. https://doi.org/10.1016/j.jveb.2016.08.071”.

Should be revised to:

“98. Wormald, D.; Lawrence, A.J.; Carter, G.; Fisher, A.D. Physiological stress coping and anxiety in greyhounds displaying inter-dog aggression. *Appl. Anim. Behav. Sci.* **2016**, *180*, 93–99. https://doi.org/10.1016/j.applanim.2016.04.007”.

“64. Wormald, D.; Lawrence, A.J.; Carter, G.; Fisher, A.D. Physiological stress coping and anxiety in greyhounds displaying inter-dog aggression. *Appl. Anim. Behav. Sci.* **2016**, *180*, 93–99. https://doi.org/10.1016/j.applanim.2016.04.007”.

Should be revised to:

“64. Wormald, D.; Lawrence, A.J.; Carter, G.; Fisher, A.D. Analysis of correlations between early social exposure and reported aggression in the dog. *J. Vet. Behav.* **2016**, *15*, 31–36. https://doi.org/10.1016/j.jveb.2016.08.071”.
